# Effect of Local Estrogen on Repairing Tympanic Membrane Perforation

**DOI:** 10.22038/ijorl.2019.39914.2343

**Published:** 2020-11

**Authors:** Behrouz Barati, Matin Ghazizadeh, Golfam Mehrparvar

**Affiliations:** *Department of Otolaryngology, Head and Neck Surgery, Shahid Beheshti University of Medical Sciences, Taleghani Hospital, Tehran, Iran.*

**Keywords:** Chronic otitis media, Estrogen, Hearing loss, Tympanoplasty

## Abstract

**Introduction:**

Tympanoplasty is a surgical treatment of tympanic membrane perforation. Many efforts have been made to increase the success rate of tympanoplasty. Some studies confirmed the positive role of estrogen in wound healing. The current study was conducted to evaluate the effect of topical estrogen on the success rate of tympanoplasty.

**Materials and Methods:**

A total of 85 patients were randomly assigned to the case and control groups. Otomicroscopic examination was performed before and 3 months following the operation. At the final stage of tympanoplasty, gelfoam was placed on the lateral side of the graft. It was soaked in dexamethasone in the control group and combination of dexamethasone and estradiol valerate solution in the case group. Hearing thresholds were measured by audiometric tests pre- and postoperatively. Hearing levels were assessed as the mean air conduction (AC) at 500, 1000, and 2000 Hz. The graft status was evaluated using otomicroscopic examination 3 months following the operation.

**Results:**

Otomicroscopic examination revealed successful graft healing in 23 of 37 and 29 of 36 patients in the control and case groups, respectively. A higher rate (80.55%) of graft repair was observed in the estradiol group, compared to that (62.16%) reported for the control group; however, the difference was not statistically significant (P=0.08). The average improvement values of the AC levels were 20.45 and 24.7 dB in the control and case groups, respectively (P=0.3). Statistical analysis among the subgroup of patients with small perforations showed that the success rate of tympanoplasty was significantly higher in the estradiol group, compared to that reported for the control group (P=0.03).

**Conclusion:**

Although topical estrogen was generally ineffective in increasing the success rate of tympanoplasty, it improved the success rate among patients with small tympanic membrane perforations.

## Introduction

Chronic tympanic membrane perforation is defined as the stable maintenance of perforation in 3 months. Chronic perforation is usually attributed to chronic inflammation in the middle ear and chronic otitis media. Unlike acute perforations, most of the chronic perforations require surgery. The main problem in the chronic tympanic membrane perforation is a lack of spontaneous healing, thereby causing the failure of epithelial growth across the perforation (1).

Chronic otitis media is a major health problem in many populations around the world. The World Health Organization estimated that 65-330 million individuals suffer from chronic otitis media (2). It is an important cause of preventable hearing loss, particularly in developing countries. The major part of the disability-adjusted life year burden must be due to hearing impairment. The chronic perforation of the tympanic membrane affects the hearing, language, social, developmental, and educational progress (3). 

The surgical treatment of tympanic membrane perforation is myringoplasty. It is defined as the simple repair of tympanic membrane perforation for the elimination of the susceptibility to middle ear infection and improvement of hearing. It helps to prevent further hearing loss which may occur in a chronically discharging ear due to the resorptive osteitis of ossicles (4). It means that the inflammatory process occurring in the middle ear causes the resorption of the ossicles due to inflammatory mediators. 

Myringoplasty is one of the most common surgical procedures conducted by otolaryngologists. Since the introduction of myringoplasty, multiple studies have been carried out on the factors affecting the outcomes, and several interventions have been tested to improve the outcomes, including the application of different techniques or use of various medications (5-11). The healing mechanism of tympanic membrane perforation has been the subject of several studies. 

The epidermis is the first layer to close a perforation due to its migratory function. The healing of the fibrous layer occurs secondarily, and the site of the response in the layer is related to the vascular distribution in the tympanic membrane. Centripetal migrating epithelium from the proliferation center plays a crucial role in the healing process (12-14).

The process of healing resembles cutaneous wound healing except that the tympanic membrane is unique in the lack of a supportive matrix beneath the regenerating epithelia. This prevents the influx of reparative cells and nutrients and usual fibroblastic reaction. 

A keratin scaffold may not be important in the healing process. Migration across the layers of the tympanic membrane appears to account for the closure of the perforation (15). Given the similarities, it seems that promoting factors in wound healing should have similar effects on the repair of tympanic membrane perforation. According to the principles of in situ tissue, the engineering regeneration of tissue requires the introduction of three basic elements, namely cells, scaffolds, and regulatory factors, under appropriate conditions (16). 

Most of the observations related to the role of estrogen in wound healing have emerged from studies carried out on the aging process. There is a substantial body of research investigating the changes that contribute to the aging process. Age-associated impaired healing correlates with increased inflammation, increased matrix proteolysis, and delayed re-epithelialization leading to chronic wound states and processes modulated by exogenous estrogen treatment (17). The supplemental use of topical estrogen has been demonstrated to improve healing in elderly women (18). Sex steroid hormones are important for controlling skin turnover. It has been shown that estrogen has a mitogenic effect on keratinocytes and increases the rate of epithelialization post-wounding in animal models. Exogenous systemic or topical estrogen treatment enhances healing by the stimulation of matrix deposition and reduction of inflammation (19). 

There have been a limited number of basic studies on the role of sex hormones in ear surgery. The potential effect of topical estrogen on tympanic membrane repair after paper patch myringoplasty has been previously investigated in two separate studies with successful results (20-21).

With this background in mind, the current study investigated whether topical estrogen could have similar effects on healing the tympanic membrane and improving the outcomes of myringoplasty.

## Materials and Methods

This prospective double-blind clinical trial was carried out to evaluate the effect of topical estrogen on the success rate of myringoplasty. The study was *approved by the Ethics Committee* of Shaheed Beheshti University of Medical Sciences in Tehran, Iran. This study was conducted on all the patients with tympanic membrane perforation who referred to the Otolaryngology Clinic of Taleghani Hospital in Tehran, Iran, within 3 years up to 2018. In addition, all the participants who enrolled in the study signed informed consent.

After taking a complete medical history and physical examination with special attention to related conditions (e.g., sinonasal disorders and craniofacial abnormalities), otomicroscopic examination and audiometric tests were performed. The size of perforation was classified as small (<50% of the tympanic membrane) or large. The patients with underlying conditions contributing to the failure of surgery according to the literature (5, 19, 22-24) were excluded from the present study. Any subjects with the following conditions did not enter the study:

- Acute tympanic membrane perforation
- Marginal perforation
- Contralateral ear pathology (i.e., perforation, retraction pocket, and myringosclerosis)
- Active otorrhea during the last 3 months
- Evidence of polyp, granulation tissue, or other observations suggestive of cholesteatoma in otomicroscopic examination
- History of myringoplasty/tympanoplasty in each ear
- Cleft palate (i.e., patent or submucosal) or history of cleft palate repair
- Obstructive pathologies of the nose or nasopharynx
- History of smoking
- Poor-controlled diabetes mellitus or immunocompromised conditions
- Patients with no consent for undergoing the surgery or entering the study

A total of 85 patients were included in the study using convenience sampling. In addition, the subjects were randomized using block randomization. The blocks included four cases, and eligible patients were operated by the senior author. Both the surgeon and patient were not aware of the use or nonuse of estradiol. A typical postauricular approach was performed, and the temporalis fascia graft was used in all cases. All the patients underwent type 1 tympanoplasty. After the placement of the graft underlay to the tympanic membrane and overlay to stapedius, the middle ear was packed with gelfoam soaked with dexamethasone (Dexadic 8 mg/2 ml Amp, Caspian Tamin Pharmaceutical Company, Rasht, Iran). The gelfoam placed on the lateral side of the graft was soaked in dexamethasone in the control group and combination of dexamethasone and estradiol valerate solution (Estraval 10 mg/1 ml Amp, Aburaihan Company, Tehran, Iran) in the case group. The ratio of dexamethasone to estradiol valerate solution was reported as 1:1. The gelfoam was indistinguishable in external appearance and was prepared by a scrub nurse who soaked gelfoam in dexamethasone or combination of dexamethasone and estradiol solution. The patients were discharged the day following the surgery. Follow-up visits were on the 7^th^ and 30^th^ days. After 12 weeks, otomicroscopic examination was undertaken for the evaluation of the graft status. Hearing thresholds were also measured using audiometric tests.

The patients were excluded if one of the following conditions occurred:

- The intraoperative observation of another pathology (i.e., polyp, cholesteatoma, and granulation tissue)
- Significant ear trauma in the follow-up period
- Lost to follow-up

**Statistical analysis**

Statistical analysis was conducted using the t-test and Chi-square test in SPSS software (version 18).

## Results

A total of 85 patients entered the study, including 39 women and 46 men. However, 12 subjects were excluded from the study (1 patient with severe head trauma and 11 subjects lost to follow-up). In this study, the patients were divided into the control (n=37) and case (n=36) groups. There was no significant difference between the two groups considering the age, gender distribution, and perforation size (Table.1).

**Table 1:** Age, gender distribution, and perforation size of two groups

	**Gender distribution**		**Age** (mean± standard deviation)	**Perforation size>50%** n (%)
	Female n (%)	Male n (%)		
Control group	19 (55.9)	18 (46.15)	39.21±14.05	11 (29.7)
Estradiol group	15 (44.1)	21 (53.84)	36.33±10.4	14 (38.8)
P-value	0.407		0.16	0.4

Successful graft healing based on otomicroscopic examination was observed in 23 and 29 patients in the control and case groups 3 months following the operation, respectively. A higher rate (80.55%) of success was observed in the estradiol group, compared to that (62.16%) reported for the control group; however, the difference was not statistically significant (P=0.08). The hearing levels were assessed as the mean air conduction at 500, 1000, and 2000 Hz, compared to the preoperative measures. 

The mean changes were 20.45 and 24.7 dB in the control and case groups, respectively (P=0.3). 

The patients’ gender and perforation size were considered the possible variables affecting the results. Therefore, the association between gender and perforation size with the success rate was separately evaluated (Table.2). Statistical analysis confirmed a significant effect of perforation size (>50% and <50% of the tympanic membrane) on the rate of successful healing (P=0.038).

**Table 2:** Potential prognostic variables

**Result**	**Variable**	**P-value**	**Odds ratio**	**95% confidence interval**
Repaired tympanic membrane	Gender	0.9	0.46	0.09-2.45
	Size of perforation	0.038	2.99	1.04-8.56
	Estradiol	0.083	2.52	0.87-7.28

A subgroup of patients with small perforations (<50% of the tympanic membrane) was separated, including 24 cases and 24 controls, and the effect of local estradiol was again evaluated among them (Table.3). The success rate of tympanoplasty in subjects with small tympanic membrane perforations was significantly higher in the estradiol group in comparison to that reported for the control group (P=0.03).

Mean changes in the hearing thresholds were calculated at 13.89 and 15.12 dB in the control and case groups among this subgroup of patients, respectively (P=0.28).

**Table 3:** Rates of graft success in patients with small perforation (<50% of tympanic membrane area)

**Result**		**Group**		
		**Control** **n (%)**	**Treatment** **n (%)**	
Repaired	Male	8 (30.7)	12 (54.5)	
	Female	9 (40.9)	11 (42.3)	
Failed	Male	3 (11.5)	1 (4.5)	
	Female	4 (15.4)	0 (0)	
Total		24	24	0.03

## Discussion

Myringoplasty is a standard surgical procedure for the treatment of chronic tympanic membrane perforation. The most common graft used for this purpose is the temporal fascia graft. The graft in myringoplasty plays the role of a scaffold for the epithelium of the perforation edges to epithelialize and close the perforation. Multiple studies have been carried out on the effect of different variables on the outcomes of myringoplasty some of which can act as confounding variables in the present study (22-25). In the current study, confounders were eliminated by the exclusion of the patients with comorbid conditions. Physiologic similarities between wound healing and repair of tympanic membrane perforation formed *the basis of the present study. This concept has also been the subject of several studies.*

*For example, b*ased on* the *promoting *role of insulin in* ulcer epithelializing and healing, a pilot study was carried out by Pujary et al. Tympanic membrane perforations made in guinea pigs. Insulin was proven to be effective on improving the epithelialization of perforation (26). Acharya et al. investigated the utility of basic fibroblast growth factor in tympanic membrane perforation. In their small cohort of pediatrics, tympanic membrane perforations were successfully closed in 7/12 (58%) and 10/12 (83%) children at the first attempt and overall, respectively (27).

The influence of estrogen on wound healing has been previously investigated in several studies. Cellular migration as an important step in both cutaneous wound healing and tympanic membrane repair is under the control of estrogen hormone (28,29). Another study examined the potential effect of topical estrogen on the repair of the tympanic membrane after paper patch myringoplasty (20.) According to the results of the aforementioned study, the patients treated with an estrogen-impinged paper patch showed a significantly higher rate of perforation closure after 30 days in comparison with those who did not receive estrogen. Seliet et al. also carried out a similar study on the perforation size of less than 40% of the tympanic membrane. They reported significant positive results not only regarding the healing of the tympanic membrane but also in the hearing level (21). 

In the present study, gelfoam soaked in estradiol valerate solution was used after type 1 tympanoplasty on the lateral side of the graft. Then, the rates of graft take were compared between the two groups 3 months following the surgery. 

Despite the higher rates of success in the estradiol group, compared to that reported for the control group, the difference was not statistically significant. This may be due to the insufficient number of patients. As the size of the tympanic membrane defect influences the success rate in myringoplasty (30-31), *the patients with* small perforation were separately* evaluated*. The analysis of this subgroup showed a significant improvement in graft healing with estradiol usage. 

## Conclusion

Although topical estrogen was generally ineffective in the improvement of the success rate of tympanoplasty, it caused a higher success rate in patients with small tympanic membrane perforations, compared with those who did not receive estrogen. It is suggested to carry out further studies with sufficient sample sizes and consideration of different variables, especially the size and site of perforation. In addition, performing complementary physiologic studies on the detection of estrogen receptors on the epithelial cells of the tympanic membrane can be helpful in this regard.

## References

[1] Seonwoo H, Kim SW, Kim J, Chunjie T, Lim KT, Kim YJ (2013). Regeneration of chronic tympanic membrane perforation using an EGF-releasing chitosan patch. Tissue Engineering Part A.

[2] Acuin J (2004). Chronic suppurative otitis media: burden of illness and management options.

[3] Monasta L, Ronfani L, Marchetti F, Montico M, Brumatti LV, Bavcar A (2012). Burden of disease caused by otitis media: systematic review and global estimates. PloS one.

[4] Dangol K, Shrivastav RP (2017). Study of Various Prognostic Factors Affecting Successful Myringoplasty in a Tertiary Care Centre. Int Arch Otorhinolaryngol.

[5] Onal K, Uguz MZ, Kazikdas KC, Gursoy ST, Gokce H (2005). A multivariate analysis of otological, surgical and patient‐related factors in determining success in myringoplasty. Clin Otolaryngol.

[6] Pinar E, Sadullahoglu K, Calli C, Oncel S (2008). Evaluation of prognostic factors and middle ear risk index in tympanoplasty. Otolaryngol Head Neck Surg.

[7] Merenda D, Koike K, Shafiei M, Ramadan H (2007). Tympanometric volume: a predictor of success of tympanoplasty in children. Otolaryngol Head Neck Surg.

[8] El-Anwar MW, El-Ahl MA, Zidan AA, Yacoup MA (2015). Topical use of autologous platelet rich plasma in myringoplasty. Auris Nasus Larynx.

[9] Sergi B, Galli J, De Corso E, Parrilla C, Paludetti G (2011). Overlay versus underlay myringoplasty: report of outcomes considering closure of perforation and hearing function. Acta Otorhinolaryngol Ital.

[10] Kamath MP, Sreedharan S, Rao AR, Raj V, Raju K (2013). Success of myringoplasty: Our experience. Indian J Otolaryngol Head Neck Surg.

[11] Lou Z (2017). FGF-2 for subacute tympanic membrane perforations. Am J Otolaryngol.

[12] Lou ZC (2013). Healing Mechanisms of Human Traumatic Tympanic Membrane Perforations. Otolaryngol Head Neck Surg.

[13] Huang P, Zhang S, Gong X, Wang X, Lou ZH (2018). Endoscopic observation of different repair patterns in human traumatic tympanic membrane perforations. Brazilian journal of otorhinolaryngology.

[14] Ahluwalia H, Narain P, Ahluwalia A, Singh J, Singh A (2018). Determinants of holistic outcome in traumatic tympanic membrane perforation. Indian J Otolog.

[15] Santa Maria PL, Redmond SL, Atlas MD, Ghassemifar R (2010). Histology of the healing tympanic membrane following perforation in rats. Laryngoscope.

[16] Hardman MJ, Ashcroft GS (2008). Estrogen, not intrinsic aging, is the major regulator of delayed human wound healing in the elderly. Genome Biol.

[17] Kanemaru S, Ito J, editor ( 2015). regeneration of tympanic membrane. Regenerative Medicine in Otolaryngology.

[18] George Broughton II, Janis JE, Attinger CE (2006). Wound healing: an overview. Plast Reconstr Surg.

[19] Pastar I, Stojadinovic O, Yin NC, Ramirez H, Nusbaum AG, Sawaya A (2014). Epithelialization in wound healing: a comprehensive review. Advances in wound care.

[20] Barati B, Abtahi SH, Hashemi SM, Okhovat SA, Poorqasemian M, Tabrizi AG (2013). The effect of topical estrogen on healing of chronic tympanic membrane perforations and hearing threshold. Journal of research in medical sciences: the official journal of Isfahan University of Medical Sciences.

[21] Seliet AM, Razik MM, Kazeem NG, Ibrahim AA, Bioumy OE (2015). The effect of topical estrogen on healing of chronic tympanic membrane perforations and hearing threshold. Benha Medical Journal.

[22] Yurttaş V, Ural A, Kutluhan A, Bozdemir K (2015). Factors that may affect graft success in tympanoplasty with mastoidectomy. ENT Updates.

[23] Ordóñez-Ordóñez LE, Angulo-Martínez ES, Prieto-Rivera JA, Almario-Chaparro JE, Guzmán-Durán JE, Lora-Falquez JG (2008). Risk factors leading to failure in myringoplasty: a case-control study. Acta Otorrinolaringol Esp.

[24] Salviz M, Bayram O, Bayram AA, Balikci HH, Chatzi T, Paltura C (2015). Prognostic factors in type I tympanoplasty. Auris Nasus Larynx.

[25] Becvarovski Z, Kartush JM (2001). Smoking and tympanoplasty: implications for prognosis and the Middle Ear Risk Index (MERI). Laryngoscope.

[26] Pujary P, Pujary K, Ramawamy B, Kanth S (2011). Topical insulin for treatment of small central perforations-a pilot study. Int Adv Otol.

[27] Acharya AN, Coates H, Tavora-Vieira D, Rajan GP (2015). A pilot study investigating basic fibroblast growth factor for the repair of chronic tympanic membrane perforations in pediatric patients. Int J Pediatr Otorhinolaryngol.

[28] Gilliver SC, Ashworth JJ, Ashcroft GS (2007). The hormonal regulation of cutaneous wound healing. Clin Dermatol.

[29] Campbell L, Emmerson E, Davies F, Gilliver SC, Krust A, Chambon P (2010). Estrogen promotes cutaneous wound healing via estrogen receptor β independent of its antiinflammatory activities. J Exp Med.

[30] Lee P, Kelly G, Mills RP (2002). Myringoplasty: does the size of the perforation matter?. Clin Otolaryngol Allied Sci.

[31] Das A, Sen B, Ghosh D, Sengupta A (2015). Myringoplasty: impact of size and site of perforation on the success rate. Indian J Otolaryngol Head Neck Surg.

